# Health Tract No. 346

**Published:** 1868-11

**Authors:** 


HEALTH TRACT NO. 346.

### Healthful Localities.

There are three ways of ascertaining the healthfulness of a place:

1st.—The average duration of life.

2d.—The death rate; or number of persons dying out of one thousand.

3d—The respective numbers which give one death a year, and by comparing them, we ascertain the relative healthiness of countries and cities.

1st.—The average duration of life in

| | | | |
|---|---|---|---|
| Russia | is 25 years. | Sweden | is 44 years. |
| Caucasus | is 25 " | Switzerland | is 45 " |
| Venetia | is 33 " | England | is 45 " |
| Holland | is 35 " | Norway | is 50 " |

2d.—There die out of every thousand of the inhabitants of

| | | | |
|---|---|---|---|
| Caucasus, | 52 | France, | 23 |
| Russia, | 36 | Belgium, | 22 |
| Italy, | 31 | England, | 22 |
| Holland, | 26 | Norway, | 17 |
| United States, | 24 | Ireland, | 16 |
| Hanover, | 23 | Ferroe Islands. | 12 |

3d.—One death a year occurs in

| | | | |
|---|---|---|---|
| Italy, in every | 28 | Russia, in every | 44 |
| Spain, " | 28 | Germany " | 45 |
| Prussia. " | 29 | Norway, " | 48 |
| France, " | 30 | Iceland, " | 53 |
| Austria, " | 30 | England, " | 58 |
| Switzerland, " | 40 | Ireland, " | 63 |

Ferroe Islands, in every............83

In cities, one die annually out of every

| | |
|---|---|
| 19 in Montreal, (1836.) | 27 in Liverpool. |
| 21 " London. | 28 " Glasgow. |
| 25 " Dublin. | 30 " Edinburgh. |
| 26 " New York. | 32 " Montreal, (1860.) |

33 in Vienna.

New York City is really one of the healthiest of the largest cities of the world, although it does not appear so in the above, because there are two elements of death not counted in other cities; the still-born and the poor emigrants reaching here dead or in a dying condition. Other cities do not count the still-born, and the emigration to New York is many times greater than that to any other city on the globe.

It will be observed that under the sunny skies of Italy, more persons die than in any other civilized country, (except Russia,) 31 in every thousand; 22 in England; 16 in Ireland; 12 in the Ferroe Islands. It is because southern Italy is an immense marshy country breeding pestilence and death, and it will continue to do so, until they re-open the drains made by the Romans. New Orleans was a nest of venomous pestilence before the war; during the war it was systematically drained and no pestilence visited it; showing that any locality may be made healthful, if well drained and kept clean.

